# A serine sensor for multicellularity in a bacterium

**DOI:** 10.7554/eLife.01501

**Published:** 2013-12-17

**Authors:** Arvind R Subramaniam, Aaron DeLoughery, Niels Bradshaw, Yun Chen, Erin O’Shea, Richard Losick, Yunrong Chai

**Affiliations:** 1 Department of Molecular and Cellular Biology, Harvard University, Cambridge, United States; 2 Faculty of Arts and Sciences Center for Systems Biology, Harvard University, Cambridge, United States; 3 Department of Chemistry and Chemical Biology, Harvard University, Cambridge, United States; 4 Howard Hughes Medical Institute, Harvard University, Cambridge, United States; 5 Department of Biology, Northeastern University, Boston, United States; National Institute of Child Health and Human Development, United States

**Keywords:** codon usage, ribosome pausing, starvation, biofilm, *B. subtilis*

## Abstract

We report the discovery of a simple environmental sensing mechanism for biofilm formation in the bacterium *Bacillus subtilis* that operates without the involvement of a dedicated RNA or protein. Certain serine codons, the four TCN codons, in the gene for the biofilm repressor SinR caused a lowering of SinR levels under biofilm-inducing conditions. Synonymous substitutions of these TCN codons with AGC or AGT impaired biofilm formation and gene expression. Conversely, switching AGC or AGT to TCN codons upregulated biofilm formation. Genome-wide ribosome profiling showed that ribosome density was higher at UCN codons than at AGC or AGU during biofilm formation. Serine starvation recapitulated the effect of biofilm-inducing conditions on ribosome occupancy and SinR production. As serine is one of the first amino acids to be exhausted at the end of exponential phase growth, reduced translation speed at serine codons may be exploited by other microbes in adapting to stationary phase.

**DOI:** http://dx.doi.org/10.7554/eLife.01501.001

## eLife digest

Bacteria use several different mechanisms to recognize and respond to changes in their environment. Protein sensors, for example, relay signals from the cell surface to target molecules within the cell. Additionally, RNA sensors can respond to internal levels of chemicals by regulating the expression of genes. Now, Subramaniam et al. have discovered a sensing mechanism in bacteria that does not rely on either protein sensors or RNA sensors.

Bacteria normally live as free swimming cells in water. But sometimes, in response to certain environmental conditions, they form a multicellular community called a biofilm. In this biofilm, bacteria encase themselves with layers of carbohydrates and proteins, which then protect the bacteria from adverse chemicals such as antibiotics.

A protein known as SinR plays a key role during biofilm formation in the model bacterium *Bacillus subtilis*. SinR normally prevents the formation of the carbohydrates and proteins that make up the biofilm. Upon the decision to form a biofilm, *B. subtilis* counters the effect of SinR by producing an anti-SinR protein called SinI.

Now Subramaniam et al. have found that as well as producing the SinI protein, *B. subtilis* use an additional mechanism to promote biofilm formation. This mechanism relies on codons, the elements within genes that correspond to specific amino acids. Six different codons correspond to the amino acid serine, and the gene for the SinR protein contains above average numbers of four of them. These four codons are highly sensitive to serine levels, and they decrease the levels of the SinR protein when there is less serine in the environment, as happens to be the case in biofilms. And since SinR prevents the production of the carbohydrates and proteins that make up the biofilms, a decrease in the levels of SinR leads to an increase in the production of biofilms.

Since the serine codons at the heart of the sensing mechanism discovered by Subramaniam et al. are present in all forms of life, from viruses to humans, it is possible that similar sensing mechanisms might be found in contexts other than bacterial biofilms, such as in viral infection and cancer.

**DOI:** http://dx.doi.org/10.7554/eLife.01501.002

## Introduction

Bacteria constantly monitor their environment and internal physiological state so that they can adapt to changing conditions. A wide variety of sensing mechanisms are deployed for this purpose, including dedicated protein sensors, such as histidine kinases, which mediate changes in gene expression by controlling the phosphorylation of cognate response regulators in response to environmental cues (West and Stock, 2001). Bacteria also sense changes in their environment and physiology by means of dedicated RNAs, such as the highly structured, leader RNA for the tryptophan operon, which controls the transcription of downstream genes in the operon by a mechanism involving ribosome stalling at tryptophan codons (Henkin and Yanofsky, 2002). Here we report the discovery of an unusually simple mechanism of environmental sensing involved in the process of biofilm formation by the bacterium *B. subtilis* that does not require a dedicated RNA or protein.

**Figure 1.**
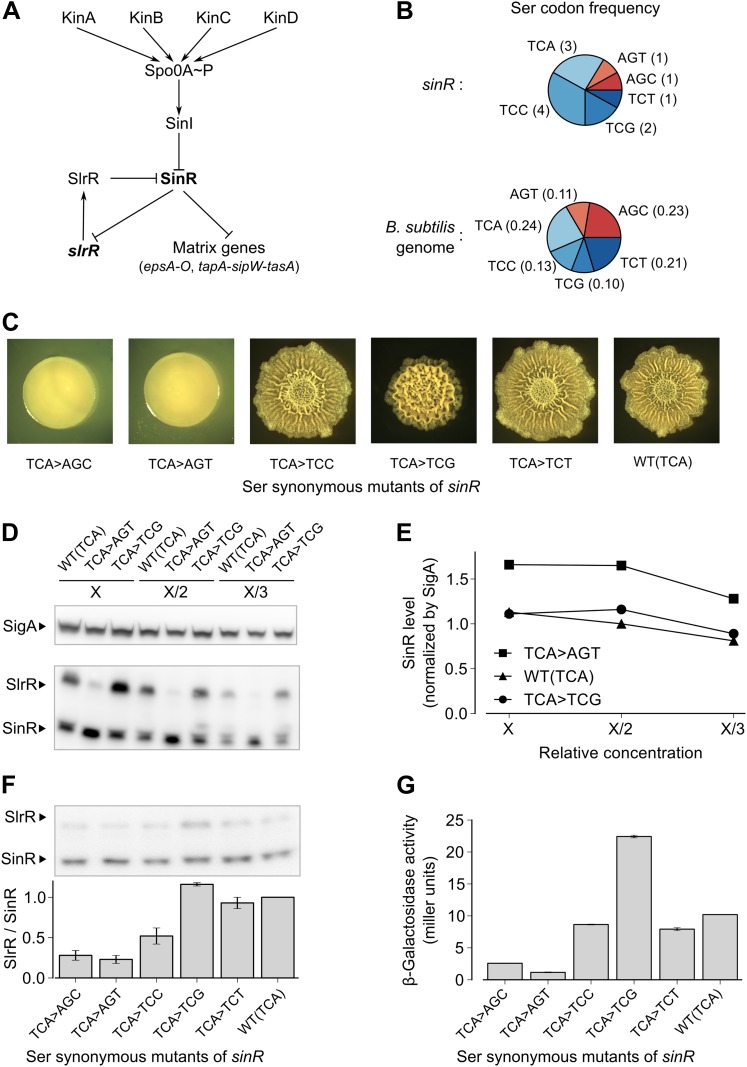
Switching synonymous serine codons in *sinR* affects biofilm formation. (**A**) Regulatory circuit controlling biofilm formation in *B. subtilis*. (**B**) Top: Serine codon usage in the *sinR* coding sequence. Number within parenthesis indicates the frequency of the corresponding codon in *sinR*. Bottom: Average serine codon usage across 4153 protein-coding sequences in the *B. subtilis* genome. Number within parenthesis indicates the relative frequency of each codon in the genome. (**C**) Colony morphology for the wild-type strain and the indicated *sinR* synonymous variants grown on solid biofilm-inducing medium. Three TCA codons in the wild-type sequence of *sinR* were switched to each of the other five serine codons. The wild-type (WT) *sinR* sequence was replaced by the *sinR* synonymous mutant at the native *sinR* locus of the strain 3610. (**D**) SinR protein level during entry into biofilm formation (OD_600_ = 2) measured using an anti-SinR antibody that also cross-reacts with SlrR, a protein that is 85% identical to SinR. Western blot against the RNA polymerase subunit SigA was used as the loading control. Whole cell lysates were loaded at different dilutions (indicated as X, X/2, and X/3). (**E**) Densitometry of SinR bands in (**D**) after normalization by SigA. (**F**) Top panel: Western blot against SinR and SlrR using anti-SinR antibody. Bottom panel: Densitometry ratio of the SlrR and SinR bands in the top panel. Error bars represent standard error over three replicate Western blots. The SlrR/SinR ratio for each blot was normalized such that the wild-type strain had a ratio of 1. (**G**) Matrix gene expression monitored using a P_*epsA*_–*lacZ* transcriptional reporter inserted at the chromosomal *amyE* locus. *β*-galactosidase activity was measured at OD_600_ = 2 in liquid biofilm-inducing medium. Error bars represent standard error of three measurements. **DOI:** http://dx.doi.org/10.7554/eLife.01501.003

Biofilm formation involves a switch from planktonic growth as individual cells to the formation of complex, multicellular communities in response to environmental cues (Kolter and Greenberg, 2006). In *B. subtilis*, these communities are embedded in a self-produced matrix consisting of polysaccharide and an amyloid-like protein, which are specified by the *epsA-O* and the *tapA-sipW-tasA* operons, respectively (Branda et al., 2001; Kearns et al., 2005). The transition to multicellularity is governed in part by four histidine kinases (KinA, KinB, KinC and KinD) that control the phosphorylation of the response regulator, Spo0A, a master regulator of post-exponential phase gene expression (Figure 1A) (Jiang et al., 2000; Vlamakis et al., 2013). Recent studies suggest that KinA and KinB respond to impaired respiration (Kolodkin-Gal et al., 2013), whereas KinC responds to membrane perturbations and KinD to unknown chemical signals (López et al., 2009; Shemesh et al., 2010; Chen et al., 2012; Beauregard et al., 2013). Once phosphorylated, Spo0A turns on *sinI*, a gene encoding a small protein antagonist of the biofilm-specific regulatory protein SinR (Molle et al., 2003; Kearns et al., 2005). SinR, which is produced constitutively, is a repressor of the matrix operons, *epsA-O* and the *tapA-sipW-tasA*, as well as other biofilm-related genes (Kearns et al., 2005; Chu et al., 2006; Chai et al., 2009). SinR is also a repressor of the gene for SlrR (Chu et al., 2008), which together with SinR sets up a self-reinforcing, double-negative feedback loop for matrix gene expression (Figure 1A) (Chai et al., 2010; Norman et al., 2013). A special feature of SinR of relevance to this investigation is that the expression of matrix genes is hypersensitive to small perturbations in the level of the protein (Chai et al., 2011). This hypersensitivity is attributed to molecular titration of SinR by SinI and cooperativity among SinR molecules bound to tandem target sequences at regulatory sites for the matrix operons (Chai et al., 2011; Chai et al., 2008).

**Figure 1—figure supplement 1.**
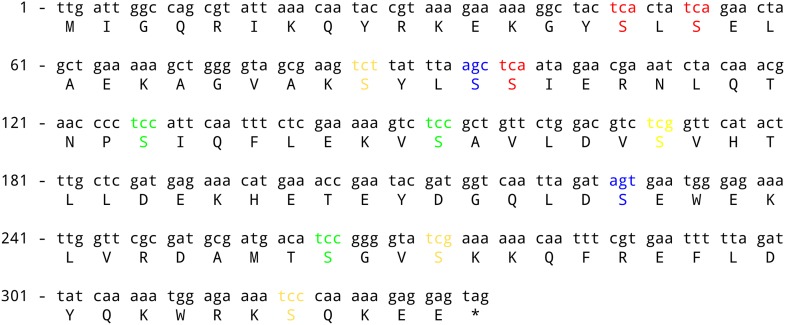
*sinR* coding sequence. The three TCA codons (switched in Figure 1) are highlighted in red. The three TCC codons and the two AGC/AGT codons (switched in Figure 1—figure supplement 2) are highlighted in green and blue respectively. The remaining serine codons are shown in yellow. **DOI:** http://dx.doi.org/10.7554/eLife.01501.004

**Figure 1—figure supplement 2.**
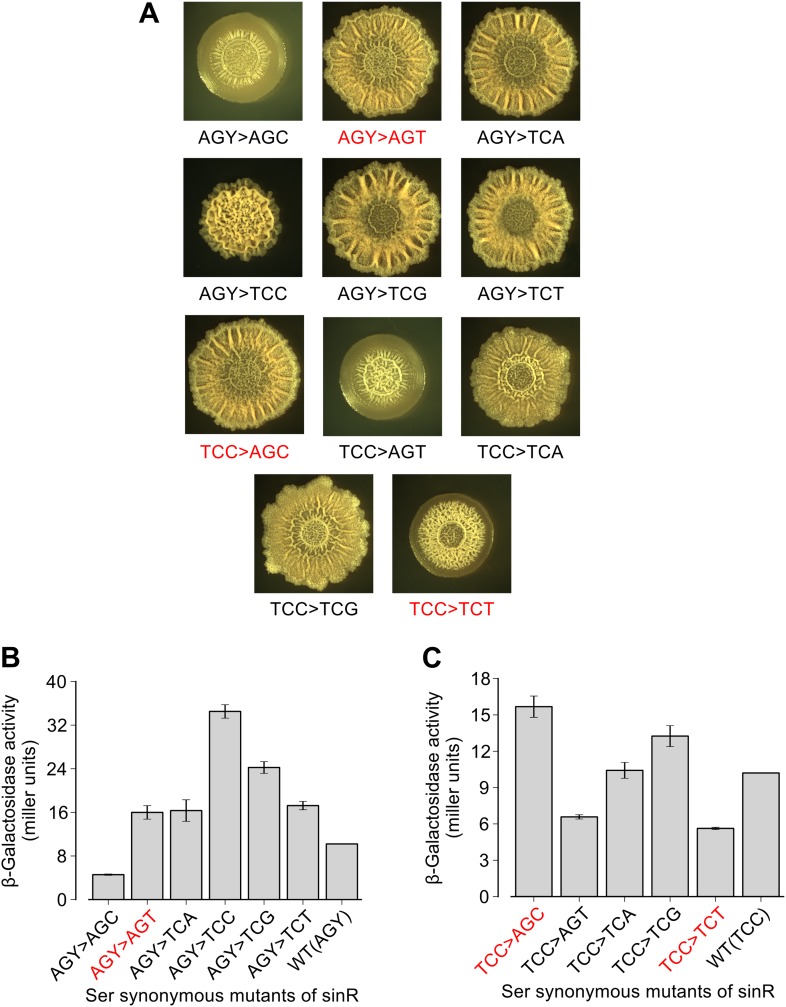
Effect of TCC and AGC/AGT synonymous substitutions in the *sinR* gene on colony morphology and biofilm reporter activity. (**A**) Colony morphology for the indicated *sinR* synonymous variants grown on solid biofilm-inducing medium. Either three TCC codons or two AGC/AGT (AGY) codons in the wild-type sequence of *sinR* were switched to remaining serine synonymous codons. The wild-type (WT) *sinR* sequence was replaced by the *sinR* synonymous variant at the native *sinR* locus of the strain 3610. Colony morphology of the wild-type strain is shown in Figure 1. (**B** and **C**) Matrix gene expression monitored using a P_*epsA*_–*lacZ* transcriptional reporter inserted at the chromosomal *amyE* locus. Strains were grown in liquid biofilm-inducing medium and *β*-galactosidase activity was measured at an OD_600_ = 2. Error bars represent standard error of three measurements. The synonymous variants highlighted in red do not follow the hierarchy between TCN and AGC/AGT codons seen for the six TCA synonymous variants in Figure 1. **DOI:** http://dx.doi.org/10.7554/eLife.01501.005

In the current study, we present evidence for the existence of a novel cellular sensing mechanism controlling biofilm formation. Rather than relying on regulation by a dedicated RNA or protein, the translation speed of ribosomes decreases at certain serine codons, resulting in lower SinR levels, which as a consequence, contributes to derepression of SinR-controlled genes. We propose that specific serine codons in the *sinR* mRNA act as a simple sensor for monitoring, and triggering a response to, serine depletion under biofilm-inducing conditions.

## Results

### Switching synonymous serine codons in *sinR* affects biofilm formation

In a genetic screen to identify suppressor mutations that rescued the biofilm-defective phenotype of a *B. subtilis* mutant (‘Materials and methods’), we unexpectedly recovered a variant that contained a ‘silent’ mutation that resulted in a switch from one serine codon to a synonymous codon in *sinR*. This observation prompted us to ask whether switching other serine codons might also influence biofilm formation. Serine is specified by six codons: AGC, AGT, TCA, TCC, TCG and TCT (where T is U in the mRNA). We noticed that the *sinR* coding sequence has a slightly higher frequency of the four TCN serine codons as compared to the average frequency of TCN codons in the *B. subtilis* genome (Figure 1B, p=0.22, N = 12). To test whether this bias towards TCN codons has an effect on biofilm formation, we systematically replaced the three TCA codons in *sinR* (Figure 1—figure supplement 1) with each of the other five serine codons. Replacing the TCA codons with AGT or AGC resulted in flat, featureless colonies on solid biofilm-inducing medium, indicating severely impaired biofilm formation (Figure 1C). In contrast, replacing the TCA codons with either TCC or TCT had little or no effect on colony morphology whereas switching to TCG increased the wrinkled appearance of the colonies (Figure 1C), which is indicative of robust biofilm formation.

Next, we asked whether switching serine codons was altering the level of SinR protein in liquid biofilm-inducing medium. Immunoblot analysis with anti-SinR antibodies revealed slightly yet consistently higher SinR levels in the strain with the AGT variant of *sinR* when compared to either the wild-type strain with three TCA codons or the TCG variant of *sinR* (Figure 1D,E).

SinR is highly similar (85% identity) to SlrR, which also plays a critical role in biofilm formation and whose gene (*slrR*) is under the direct negative control of SinR (Chu et al., 2008). Because SlrR cross reacts with the anti-SinR antibodies, we were also able to detect SlrR in our immunoblot analysis. Strikingly, the levels of SlrR were almost perfectly anti-correlated with those of SinR, with the differences in the SlrR protein levels among the *sinR* synonymous variants being much higher than the corresponding differences in SinR protein levels (Figure 1F). Because repression by SinR is ultrasensitive to SinR levels (Chai et al., 2011), small differences in SinR protein levels among *sinR* synonymous variants might be sufficient to cause large differences in the levels of expression of SinR-repressed genes such as *slrR*. Consistent with this idea, an *eps-lacZ* transcriptional fusion reporter for the SinR-repressed *epsA-O* matrix operon showed that the four TCN *sinR* variants had 3- to 19-fold higher *β*-galactosidase activity than the AGT and AGC variants (Figure 1G).

To test the generality of the observed hierarchy between the synonymous variants of *sinR*, we generated an additional set of eleven *sinR* synonymous variants in which we replaced either three TCC codons or two AGC/AGT codons (Figure 1—figure supplement 1) with their synonymous counterparts. Eight of these variants conformed to the hierarchy described above, namely, the four TCN variants behaved oppositely to the two AGC/AGT variants in colony morphology and in *eps-lacZ* reporter expression (Figure 1—figure supplement 2). The three variants that did not conform to the hierarchy could potentially reflect alterations to the mRNA sequence context near the mutation rather than the effect of a synonymous substitution per se. Taken together, the above results suggest that serine synonymous codons in the *sinR* coding sequence have a stereotypical effect on biofilm formation that is primarily determined by the differential usage of the four TCN and the two AGC/AGT codons.

### Entry into biofilm formation is accompanied by codon-specific increase in ribosome density

What is the mechanism by which serine codon usage affects SinR protein levels and biofilm formation? Synonymous codon changes can alter the synthesis of the encoded protein through changes in the translation initiation rate, mRNA levels or the ribosome elongation rate (Plotkin and Kudla, 2010). However, the effect of synonymous codon usage on the initiation rate and mRNA levels is context-specific; only codons near the AUG start site affect translation initiation (Kudla et al., 2009), whereas only codons that are located in certain regions of secondary structure or at ribonuclease cleavage sites affect mRNA levels (Bernstein et al., 2002). Our observation that synonymous codon replacements at multiple locations along *sinR* have a stereotypical effect on biofilm formation argues against such context-specific mechanisms (except for the three exceptional cases noted above).

To test the alternative hypothesis that serine codon usage might alter the ribosome elongation rate, and given that the ribosome elongation rate at a codon varies inversely with the average ribosome density at that codon, we measured ribosome density on mRNAs at single-codon resolution using the ribosome profiling method (Ingolia et al., 2009; Oh et al., 2011; Ingolia et al., 2012). We grew *B. subtilis* in liquid biofilm-inducing medium, harvested cells either during exponential phase growth (OD_600_ = 0*.*6) or during stationary phase when biofilm formation is induced (OD_600_ = 1*.*4), and performed deep-sequencing of ribosome protected mRNA fragments and size-matched total mRNA fragments. Ribosome profiling yielded 3.75 and 2.55 million sequencing reads aligning to annotated protein-coding sequences for the exponential phase sample and the biofilm entry sample, respectively. The number of reads aligning to a single codon on individual mRNAs was too low for accurate quantification of ribosome density, and was not sufficient to directly detect increased ribosome density on the *sinR* transcript. However, we reasoned that the global pattern of ribosome density at codons across all mRNAs should reflect the ribosome density on individual transcripts such as *sinR*. Therefore, we calculated the median ribosome density at each of the 61 sense codons across the 1556 protein-coding sequences in the exponential phase sample and the 1148 sequences in the biofilm entry sample that had an average coverage greater than one sequencing read per codon (Figure 2—figure supplement 1, ‘Materials and methods’). This analysis reproduced the previous observation (Li et al., 2012) of increased ribosome density 8 to 11 nt downstream of Shine-Dalgarno-like trinucleotide sequences both during exponential phase and during biofilm entry (Figure 2—figure supplement 2).

**Figure 2.**
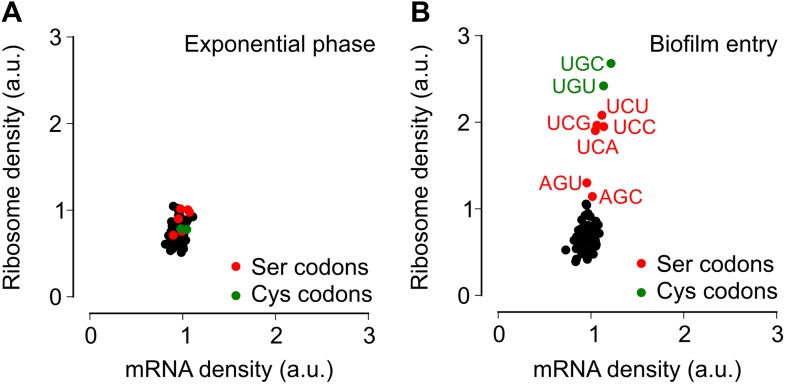
Entry into biofilm formation is accompanied by codon-specific increase in ribosome density. Genome-wide median ribosome density and total mRNA density at 61 sense codons (excluding start and stop codons) (**A**) during exponential phase growth (OD_600_ = 0.6), and (**B**) during stationary phase when biofilm formation is induced (OD_600_ = 1.4). The six serine (red) and two cysteine (green) codons are highlighted. Genome-wide ribosome density and total mRNA density were measured by deep-sequencing of ribosome-protected mRNA fragments and total mRNA fragments respectively, of a *B. subtilis* 3610 derivative (Δ*epsH*) grown in liquid biofilm-inducing medium. **DOI:** http://dx.doi.org/10.7554/eLife.01501.006

During exponential phase, median ribosome density varied over a twofold range, with no systematic difference between serine codons and the remaining codons (Figure 2A). By contrast, during biofilm entry, median ribosome density was significantly higher at serine and cysteine codons as compared to the remaining codons (Figure 2B), suggesting that the translation speed of ribosomes is selectively reduced at these codons during biofilm entry. Notably, the ribosome density at serine codons was not uniform: the four UCN codons had 1.9 to 2.1-fold higher ribosome density whereas the AGC and AGU codons had only 1.1 and 1.3-fold higher ribosome density respectively, relative to the median value across 61 sense codons. Further, this difference in ribosome density between UCN codons and the AGC/AGU codons was essentially identical when computed separately for codons located in the first half or in the second half of each gene (Figure 2—figure supplement 3), a finding that underscores the statistical robustness and the context independence of the observed difference.

**Figure 2—figure supplement 1.**
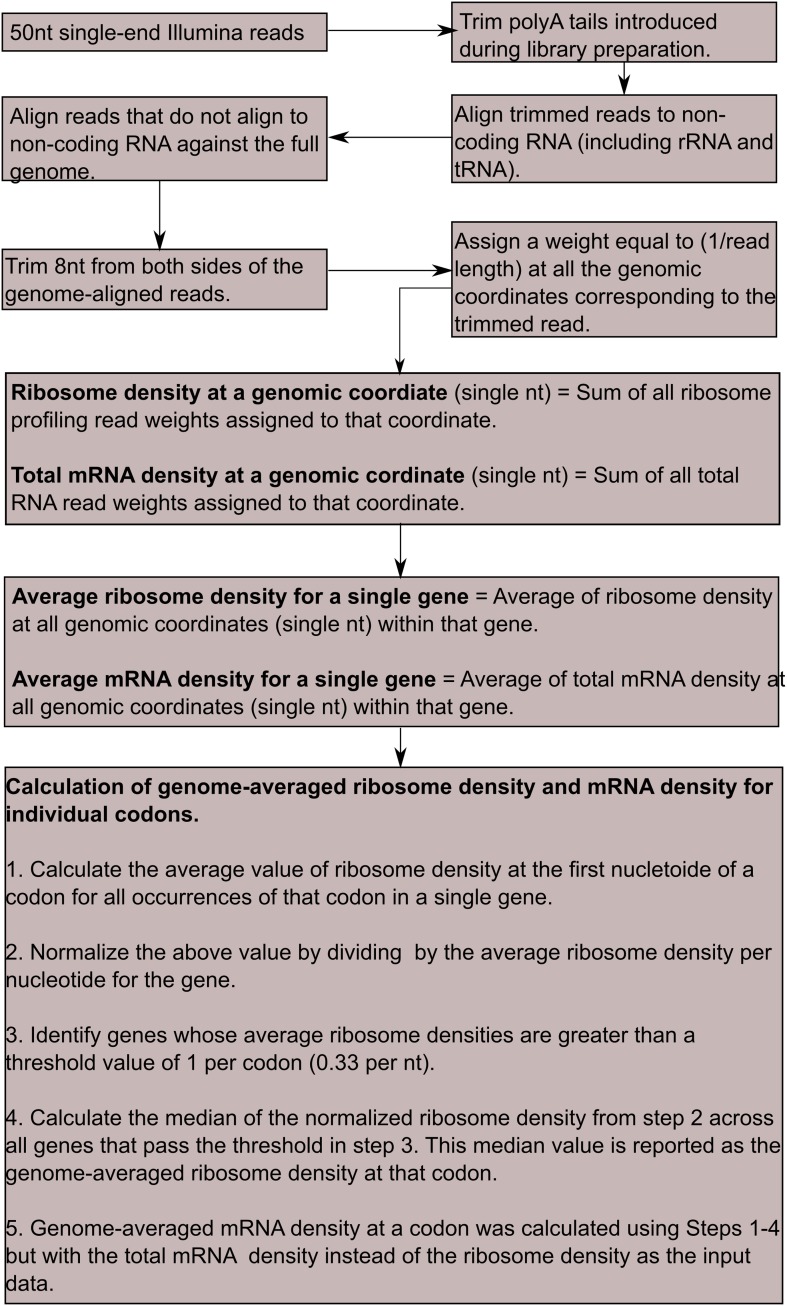
Computational workflow for deep-sequencing data analysis. All steps outlined here were performed in Bash and Python programming languages. For further details on individual steps, see ‘Materials and methods’. **DOI:** http://dx.doi.org/10.7554/eLife.01501.007

**Figure 2—figure supplement 2.**
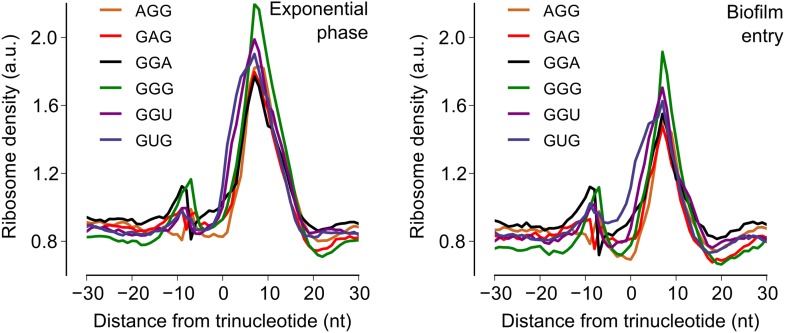
Increase in ribosome density downstream of Shine-Dalgarno-like trinucleotide sequences. Median ribosome density across all protein coding sequences was computed for the 60 nt region around each of six Shine-Dalgarno-like trinucleotide sequences (Li et al., 2012) for the exponential phase sample (left-hand panel) and the biofilm entry sample (right-hand panel). **DOI:** http://dx.doi.org/10.7554/eLife.01501.008

**Figure 2—figure supplement 3.**
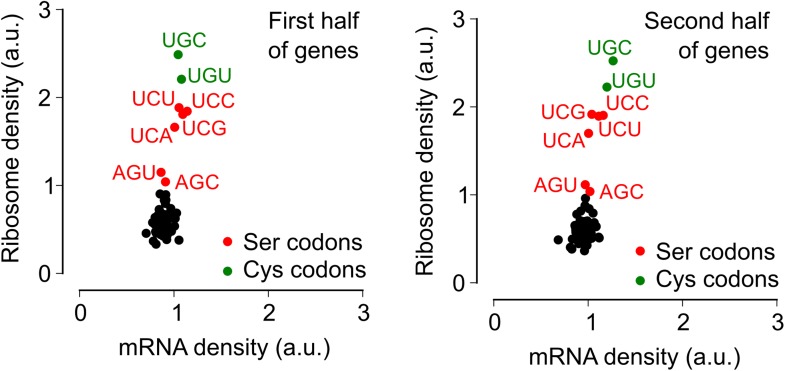
Context independence of ribosome and mRNA densities during biofilm formation. Each gene was conceptually divided into two equal halves and the ribosome density and mRNA density was computed separately for codons located either in the first half (left-hand panel) or in the second half (right-hand panel) of each gene. All other analysis steps were identical to those in Figure 2. **DOI:** http://dx.doi.org/10.7554/eLife.01501.009

### Serine starvation reduces translation speed and inhibits SinR synthesis in a codon-specific manner

**Figure 3.**
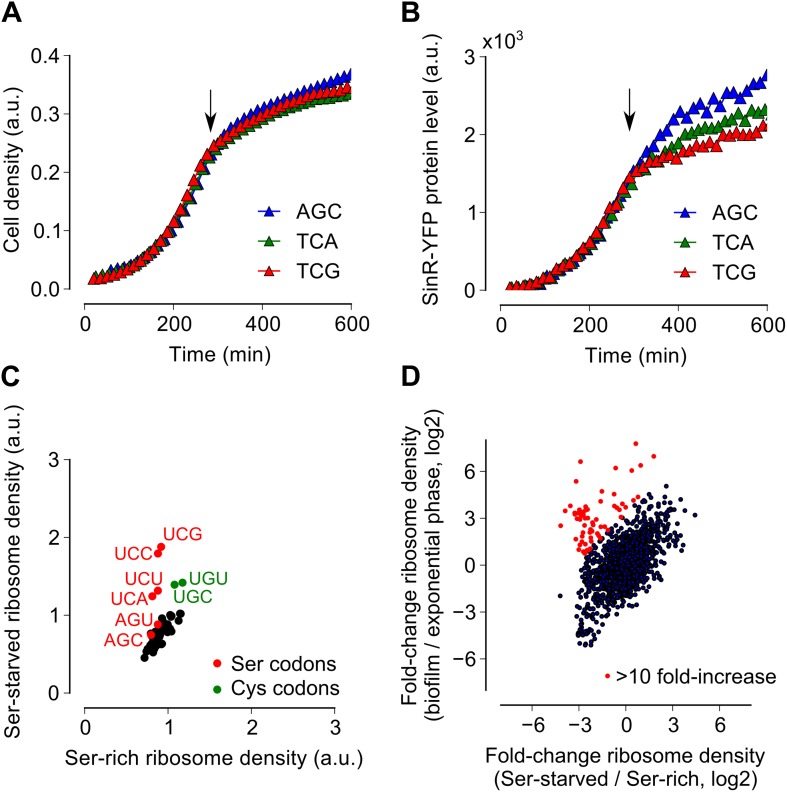
Serine starvation reduces translation speed and inhibits SinR synthesis in a codon-specific manner. (**A** and **B**) Three *sinR* synonymous variants were synthesized with 10 serine codons switched to AGC, TCA or TCG. The variants were expressed as SinR-YFP fusions from the *amyE* locus under the control of a *lac* promoter in a 3610-Δ*serA* serine auxotroph strain growing in serine-limited medium. Black arrow around 300 min indicates the onset of serine starvation caused by depletion of exogenously-added serine in the growth medium. Cell density (**A**) and the corresponding SinR-YFP protein level (**B**) were monitored using a 96-well spectrophotometer. (**C**) Genome-wide median ribosome density for 61 sense codons (excluding start and stop codons) during serine starvation (vertical axis) and serine-rich growth (horizontal axis) of a serine auxotrophic strain. (**D**) Fold-change in average ribosome density for individual genes upon biofilm entry (vertical axis) or serine starvation (horizontal axis). Genes that were preferentially up-regulated at least 10-fold upon biofilm entry in comparison to serine starvation are highlighted in red (68 genes, Table 1). Only genes with a minimum of 100 ribosome profiling reads in at least one of the samples were included in this analysis (1724 genes) and the reported log2 fold-changes are median-subtracted values across this gene set. **DOI:** http://dx.doi.org/10.7554/eLife.01501.010

Because ribosomes density increased at serine and cysteine codons only during biofilm formation and not during exponential phase growth, we hypothesized that the increased ribosome density was caused by the depletion of intracellular pools of these two amino acids during biofilm entry rather than by any intrinsic feature of the mRNA (Li et al., 2012) or the nascent polypeptide (Charneski and Hurst, 2013). Our hypothesis is also supported by the previous observation that synonymous codon usage can have a starvation-specific effect on protein levels (Subramaniam et al., 2013). Further, serine is a precursor metabolite for the biosynthesis of cysteine (Gagnon et al., 1994); hence cysteine depletion was likely the result of a decrease in intracellular serine concentration. Consistent with this hypothesis, we also observed an increase in ribosome density at both serine and cysteine codons during serine starvation of a *B. subtilis* serine-auxotrophic mutant (Figure 3C). Importantly, serine starvation resulted in an increase in ribosome density at only the four UCN serine codons but not at the AGC and AGU codons, matching the hierarchy seen during biofilm entry (Figure 2B). Serine starvation also resulted in differential levels of production of SinR-YFP protein fusions bearing different synonymous serine codons, whereas serine-rich growth resulted in identical levels of fusion protein production from these variants (Figure 3A,B). Finally, the addition of excess serine or cysteine (but not any of the other 18 amino acids) blocked biofilm formation in wild type cells as judged after 48 hr of growth in biofilm-inducing medium (Figure 3—figure supplement 1, and data not shown).

**Figure 3—figure supplement 1.**
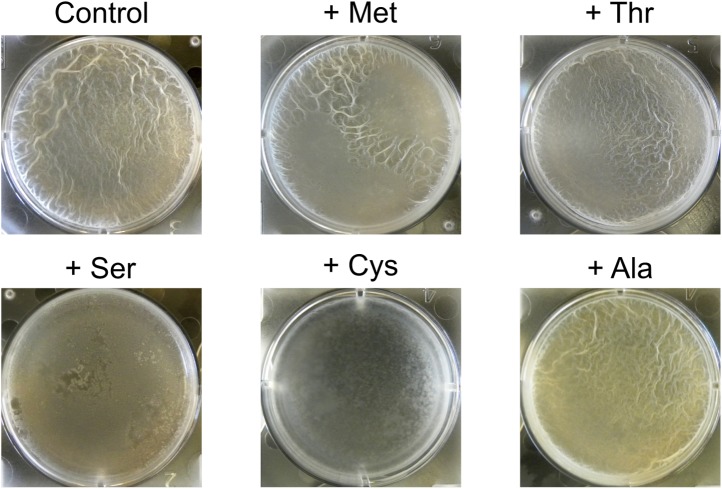
Addition of excess serine or cysteine blocks pellicle formation by *B. subtilis*. Single amino acids were added at 300 µg ml^−1^ to liquid MSgg medium. Biofilm formation of 3610 was assayed visually by pellicle formation at the air-liquid interface 48 hr after inoculation. Serine and cysteine were found to block pellicle formation out of all 20 amino acids tested. **DOI:** http://dx.doi.org/10.7554/eLife.01501.011

Given that both biofilm entry and serine starvation resulted in increased ribosome density at serine and cysteine codons, we asked whether these two apparently different conditions invoke the same gene expression program in *B. subtilis*. The fold-change in average ribosome density of *B. subtilis* genes was positively correlated between biofilm entry and serine starvation (Figure 3D, *R*^*2*^ = 0*.*27*,* p=10^−5^, 1724 genes). However, a subset of 68 genes was induced at least 10-fold higher upon biofilm entry than during serine starvation (indicated by red markers in Figure 3D, Table 1). This subset included genes for anaerobic metabolism such as *lctEP*, *nasDE*, and *cydAB*, and is consistent with the recently proposed role of impaired respiration in biofilm formation (Kolodkin-Gal et al., 2013). We observed that sulfur metabolism genes were also enriched in this subset, possibly indicating a stronger response to cysteine depletion during biofilm entry than during serine starvation.

**Table 1.** *B. subtilis* genes that have greater than 10-fold difference in expression ratio between biofilm formation and serine starvation **DOI:** http://dx.doi.org/10.7554/eLife.01501.012

Gene	A	B	C	D	E	F	Function
*albA*	9.35	−0.69	32	10129	175	144	antilisterial bacteriocin (subtilosin) production protein
*alsD*	6.38	0.95	34	1384	53	135	alpha-acetolactate decarboxylase
*alsS*	6.96	1.79	76	4613	86	396	acetolactate synthase
*cah*	2.08	−2.74	2531	5254	1486	295	S-deacylase
*ctc*	3.78	−0.73	327	2206	322	257	50S ribosomal protein L25
*cydA*	5.36	−3.18	234	4720	408	60	cytochrome bd ubiquinol oxidase subunit I
*cydB*	9.6	−1.88	9	3374	129	46	cytochrome bd ubiquinol oxidase subunit II
*cysK*	1.52	−2.21	15760	22120	8991	2580	cysteine synthase
*gcvPA*	0.97	−2.38	1500	1440	1001	255	glycine dehydrogenase subunit 1
*gcvPB*	1.05	−2.28	2029	2057	1224	335	glycine dehydrogenase subunit 2
*gspA*	4.54	−0.75	111	1261	135	106	glycosyl transferase (general stress protein)
*iseA*	2.24	−1.14	1117	2579	2521	1516	inhibitor of cell-separation enzymes
*lctP*	6.62	−2.9	62	3009	247	44	L-lactate permease
*ldh*	3.47	−3.88	2810	15,254	1732	157	L-lactate dehydrogenase
*maeN*	4.08	−1.61	148	1226	347	151	Na+/malate symporter
*mccA*	3.23	−2.72	759	3494	386	78	cystathionine beta-synthase
*metE*	2.72	−2.13	33,027	106473	32,231	9760	5-methyltetrahydropteroyltriglutamate/homocysteine S-methyltransferase
*mgsR*	4.72	−1.53	163	2099	202	93	stress transcriptional regulator
*mtlA*	3.92	−0.22	126	934	104	119	PTS system mannitol-specific transporter subunit IICB
*mtnA*	1.84	−2.79	2522	4425	3501	671	methylthioribose-1-phosphate isomerase
*mtnD*	1.72	−1.63	4095	6600	3182	1361	acireductone dioxygenase
*mtnK*	2.54	−2.64	4290	12,196	5866	1250	methylthioribose kinase
*nasD*	6.21	−0.65	217	7872	635	536	assimilatory nitrite reductase subunit
*nasE*	6.05	0.38	34	1106	63	109	assimilatory nitrite reductase subunit
*rbfK*	3.74	−2.57	1329	8681	434	97	RNA-binding riboflavin kinase
*sboA*	7.77	0.64	121	12,895	256	530	subtilosin A
*sboX*	8.26	0.19	21	3128	76	115	bacteriocin-like product
*ssuA*	2.09	−2.3	3691	7682	877	236	aliphatic sulfonate ABC transporter binding lipoprotein
*ssuB*	2.57	−2.09	2416	7004	779	242	aliphatic sulfonate ABC transporter ATP-binding protein
*ssuC*	2.17	−2.16	3784	8362	692	206	aliphatic sulfonate ABC transporter permease
*ssuD*	2.2	−2.19	12,961	29,135	2812	817	alkanesulfonate monooxygenase
*tcyJ*	3.25	−3.26	1046	4856	427	59	sulfur-containing amino acid ABC transporter binding lipoprotein
*tcyK*	3.79	−3.55	2815	19,087	1095	124	sulfur-containing amino acid ABC transporter binding lipoprotein
*tcyL*	3.33	−3.05	855	4223	387	62	sulfur-containing amino acid ABC transporter permease
*tcyM*	3.54	−2.97	1859	10,556	581	99	sulfur-containing amino acid ABC transporter permease
*tcyN*	3.38	−2.74	3363	17,149	1119	223	sulfur-containing amino acid ABC transporter ATP-binding protein
*ureA*	4.36	0.78	124	1252	77	176	urease subunit gamma
*ycgL*	0.99	−3.01	512	500	278	46	hypothetical protein
*ycgM*	2.46	−1.35	39	105	178	93	proline oxidase
*ycgN*	2.45	−1.6	928	2475	1267	556	1-pyrroline-5-carboxylate dehydrogenase
*ycnJ*	0.75	−2.63	168	138	121	26	copper import protein
*ydaG*	4.14	0.49	70	604	80	150	general stress protein
*ydbL*	3.07	−0.31	294	1210	130	139	hypothetical protein
*yeaA*	1.34	−2.59	112	138	139	31	hypothetical protein
*yezD*	2.52	−4.17	145	406	340	25	hypothetical protein
*yitJ*	3.01	−2.43	2785	10,990	4687	1153	bifunctional homocysteine S-methyltransferase/5,10-methylenetetrahydrofolate reductase
*yjbC*	3.67	−0.57	104	647	248	222	thiol oxidation management factor; acetyltransferase
*yjnA*	1.5	−2.81	972	1342	790	149	hypothetical protein
*yoaB*	2.28	−2.26	2206	5244	2862	792	negatively charged metabolite transporter
*yoaC*	2.92	−1.89	1200	4459	1436	513	hydroxylated metabolite kinase
*yrhB*	2.92	−2.93	3806	14,096	1904	333	cystathionine beta-lyase
*yrrT*	2.97	−3.12	546	2089	443	68	AdoMet-dependent methyltransferase
*ytlI*	1.66	−3.2	206	318	141	20	LysR family transcriptional regulator
*ytmI*	3.34	−3.27	3452	17,173	1640	226	N-acetyltransferase
*ytmO*	3.4	−2.88	3866	20,007	1179	213	monooxygenase
*ytnI*	3	−2.6	3522	13,770	867	189	redoxin
*ytnJ*	3.14	−2.86	10,645	45,997	2495	456	monooxygenase
*ytnL*	3.56	−2.45	1281	7371	354	86	aminohydrolase
*ytnM*	3.5	−2.6	4542	25,202	1264	277	transporter
*yuaF*	1.89	−1.74	87	157	158	63	membrane integrity integral inner membrane protein
*yvzB*	0.95	−2.48	125	118	168	40	flagellin
*yxaL*	3.54	−0.41	1069	6077	442	442	membrane associated protein kinase
*yxbB*	3.7	−0.01	108	685	118	155	S-adenosylmethionine-dependent methyltransferase
*yxeK*	0.86	−2.72	2702	2406	1073	216	monooxygenase
*yxeL*	1.29	−2.94	437	525	202	35	acetyltransferase
*yxeM*	0.87	−2.57	3003	2692	1047	233	ABC transporter binding lipoprotein
*yxeP*	1.75	−2.24	2577	4246	736	207	amidohydrolase
*yxjH*	2.02	−1.82	4162	8243	3811	1432	methyl-tetrahydrofolate methyltransferase

A—median-subtracted log2 fold-change: biofilm/exponential-phase, B—median-subtracted log2 fold-change: serine starvation/serine rich, C—raw counts: biofilm entry, D—raw counts: exponential phase, E—raw counts: serine rich, F—raw counts: serine starvation.

*In toto*, the results with the serine auxotroph support the inference that ribosome stalling observed during biofilm formation is due to a drop in intracellular serine levels. Our efforts to measure intracellular serine levels directly during growth in minimal, biofilm-inducing medium (MSgg) have been unsuccessful. Hence, we cannot rule out the less likely possibilities that biofilm entry causes serine and cysteine to be sequestered away from protein synthesis or that aminoacylation rate of the corresponding tRNAs decreases without changes in the intracellular serine and cysteine pools.

### Serine codon bias in biofilm-regulated genes reflects their expression during serine starvation

**Figure 4.**
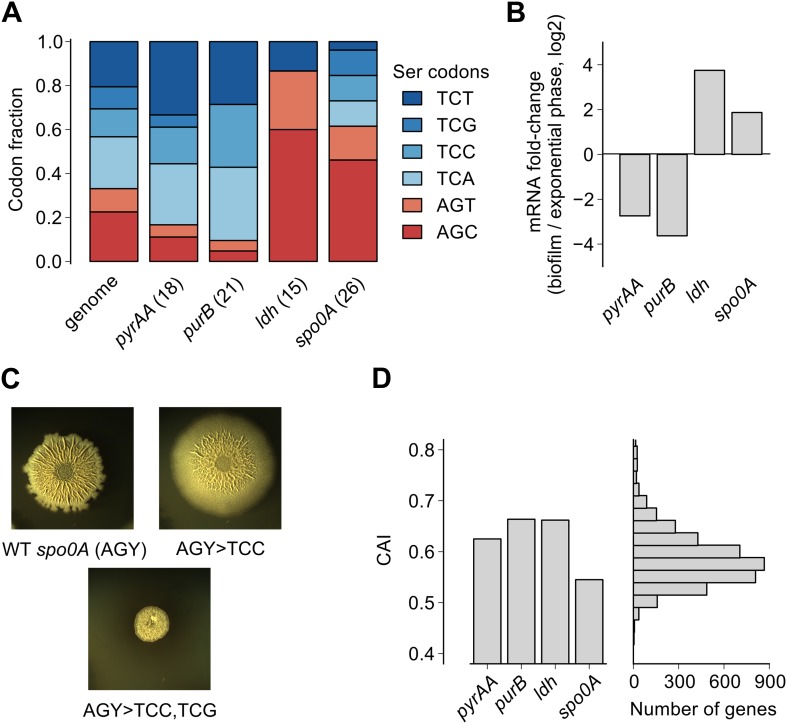
Serine codon bias of biofilm-regulated genes reflects their expression under serine starvation. (**A**) Relative serine codon fraction in genes for nucleotide biosynthesis (*pyrAA*, *purB*), lactate dehydrogenase (*ldh*) and a sporulation regulator (*spo0A*). Numbers in parentheses indicate the number of serine codons in each gene. Relative fraction of serine codons across the *B. subtilis* genome is shown for comparison. (**B**) Fold-change (expressed in log2 units) in average ribosome density upon biofilm entry for the four genes shown in **A**. (**C**) Colony morphology of a wild-type strain and two *spo0A* synonymous variants grown on solid biofilm-inducing medium. Seven AGC/AGT codons in wild-type *spo0A* were replaced by either 7 TCC codons or 3 TCC and 4 TCG codons and inserted at the chromosomal *spo0A* locus. Both the wild-type *spo0A* and the synonymous *spo0A* variants were inserted with a downstream selection marker. (**D**) Left: Codon Adaptation Index (CAI) for the four genes shown in **A**. Right: Distribution of CAI values for 4153 protein-coding sequences of *B. subtilis*. **DOI:** http://dx.doi.org/10.7554/eLife.01501.013

Here we found that the translation speed of ribosomes decreases at UCN serine codons and thereby modulates production of SinR and entry into biofilm formation. Based on our genome-wide measurements of ribosome density, we expect that the translation speed of ribosomes should decrease during biofilm entry not only on the *sinR* mRNA, but also on other mRNAs that are enriched for any of the four UCN codons. For example, we found that the four TCN codons are over-represented in two nucleotide biosynthesis genes, *pyrAA* and *purB* (Figure 4A). These two genes are also transcriptionally down regulated during biofilm entry (Figure 4B). A high frequency of TCN codons in these genes might serve to reinforce their transcriptional down regulation by reducing translation speed. Conversely, TCN codons are under-represented in the genes encoding lactate dehydrogenase, *ldh* (Cruz Ramos et al., 2000) and a master regulator of post-exponential phase gene expression, *spo0A* (Molle et al., 2003) (Figure 4A). The two genes are transcriptionally up regulated upon biofilm entry (Figure 4B). The low frequency of TCN codons in these genes might represent a mechanism for optimizing the production of their protein products by minimizing the slowing down of translation during biofilm entry. Consistent with this idea, replacement of AGC/AGT codons by TCN codons in *spo0A*, whose protein product positively regulates biofilm entry (by turning on the synthesis of the SinR antagonist SinI), resulted in defective biofilm formation (Figure 4C) in contrast to the stimulatory effect of replacing AGC/AGT codons with TCN codons in *sinR* (Figure 1—figure supplement 2).

## Discussion

Together, our results implicate serine depletion as an environmental cue that contributes to promoting biofilm formation in *B. subtilis* together with other cues that are sensed by the histidine kinases KinA–D. Serine depletion is sensed through a remarkably simple mechanism based on reduced translation speed at UCN serine codons in the mRNA for a regulatory protein, SinR, whose repressive effects are highly sensitive to small changes in the level of the protein. We presume that UCN codons lower SinR levels simply by slowing the rate of ribosome movement along the mRNA (elongation). However, it is possible that the reduced translation speed at UCN codons during biofilm entry could be followed by downstream events such as ribosome rescue (Keiler et al., 1996) or mRNA decay (Hayes and Sauer, 2003) that might also contribute to lowering the levels of the SinR protein.

The serine sensing mechanism uncovered here operates through over-representation of the TCN serine codons in the *sinR* gene without the necessity for any other dedicated protein or RNA for sensing serine depletion. By contrast, transcriptional attenuation, a widespread mechanism among bacteria for sensing amino acids that also relies on changes in translation speed, involves a translation-transcription coupling mechanism that is mediated by highly structured mRNAs and leader peptides (Henkin and Yanofsky, 2002).

We note that the biased usage of the four TCN serine codons, which act as starvation sensors during biofilm formation, is not evident from widely-used phenomenological measures of codon bias such as the codon adaptation index (Figure 4D), which primarily reflects codon preferences during exponential growth (Sharp and Li, 1987; Andersson and Kurland, 1990). The difference in translation speed between the four UCN codons and the two AGC/AGU codons under biofilm-inducing conditions is likely mediated by differences in concentration of the corresponding aminoacylated tRNAs (Elf et al., 2003; Dittmar et al., 2005), as was recently observed in serine-starved *E. coli* (Subramaniam et al., 2013). Interestingly, the hierarchy between UCN codons and AGC/AGU codons in *B. subtilis* during serine starvation is similar to the one in *E. coli* even though copy numbers of the corresponding tRNA genes have diverged significantly between these two organisms (Lowe and Eddy, 1997). Despite different tRNA gene copy numbers, it is possible that the relative abundances of the serine tRNA isoacceptors are similar between the two organisms or that their relative abundances might be regulated in the same manner in response to nutrient deprivation (Doi et al., 1966).

Serine is one of the first amino acids to be completely consumed from the culture medium when either *B. subtilis* or *E. coli* cells are grown in complex rich medium (Liebs et al., 1988; Prüss et al., 1994; Sezanov et al., 2007). Indeed, increased ribosome density has been observed at serine codons during growth of *E. coli* in Luria-Bertani broth (Li et al., 2012). Thus the role of synonymous serine codons as starvation sensors discovered here in the specific context of biofilm formation might be a general regulatory strategy in microbes for adapting to nutrient depletion at the end of exponential phase growth. It is noteworthy that depletion of specific amino acids affects developmental transitions in several eukaryotic cells (Marin, 1976; Sundrud et al., 2009; Wang et al., 2009). It will be interesting to test whether a codon-based sensing mechanism, similar to the one found here in bacterial biofilm development, also plays a role in eukaryotic cells during amino acid depletion.

## Materials and methods

### Bacterial strains and media

For ribosome profiling during biofilm formation, a 3610-Δ*epsH* strain (RL3852) was used to ensure dispersed growth in liquid media (Kearns et al., 2005). For serine starvation experiments, a serine-auxotrophic 3610-Δ*serA* strain (YC865) was used. A list of strains, plasmids, and oligonucleotides used in this work are summarized in Supplementary file 1.

For general purposes, *B. subtilis* strains PY79, 3610, and their derivatives were grown in Luria-Bertani (LB) medium (10 g tryptone, 5 g yeast extract, and 5 g NaCl per liter broth) at 37°C. Antibiotics were added to the media at the following concentrations for *B. subtilis* strains: 10 µg ml^−1^ of tetracycline, 100 µg ml^−1^ of spectinomycin, 10 µg ml^−1^ of kanamycin, 5 µg ml^−1^ of chloramphenicol, and 1 µg ml^−1^ of erythromycin. Minimal MSgg medium was used as the liquid biofilm-inducing medium. The same medium with 1.5% Bacto-agar (Difco, Franklin Lakes, NJ) was used as the solid biofilm-inducing medium. MSgg medium composition: 5 mM potassium phosphate (pH 7), 100 mM MOPS (pH 7), 2 mM MgCl_2_, 700 µM CaCl_2_, 50 µM MnCl_2_, 50 µM FeCl_3_, 1 µM ZnCl_2_, 2 µM thiamine, 0.5% glycerol, 0.5% glutamate, 50 µg ml^−1^ tryptophan, 50 µg ml^−1^ phenylalanine and 50 µg ml^−1^ threonine. For overnight growth of serine auxotrophic 3610 strains, MSgg medium was supplemented with serine to a final concentration of 5 mM. For serine starvation experiments in which YFP fluorescence was measured (Figure 3A,B), MSgg medium was supplemented with 800 µM serine and 400 µM serine methyl-ester (Sigma, St. Louis, MO). Serine methyl-ester was added to ensure slow growth under serine starvation conditions.

### Strain construction

General methods for molecular cloning followed published protocols (Sambrook 2001). SPP1 phage-mediated transduction was used to transfer antibiotic-marked DNA fragments between different strains (Kearns et al., 2005). Long-flanking PCR mutagenesis was applied to generate insertional deletion mutations (Wach 1996). Synonymous switches in *sinR* and *spo0A* were generated by using synthetic DNA fragments (Genewiz, South Plainfield, NJ) or by applying site-directed mutagenesis (Roche, Switzerland). Sequences of the primers used in constructing mutant *sinR* alleles are described in Supplementary file 1. Incorporation of synonymous substitutions into the *sinR* or *spo0A* gene at the native locus was done by allele exchange and followed a method described previously (Chai et al., 2011).

### Biofilm assays

*B. subtilis* cells were first grown in LB broth at 37°C to mid-exponential phase. For formation of biofilm colonies, 2 µl of the cells was spotted onto MSgg medium solidified with 1.5% agar. Plates were incubated at 23°C for 3–4 days before analysis. All images were taken using either a Nikon CoolPix 950 digital camera or using a SPOT camera (Diagnostic Instruments, Sterling Heights, MI). Assays for the *β*-galactosidase activities were described previously (Kearns et al., 2005).

### Genetic screen for suppressor mutants

Following a previously-published protocol (Chai et al., 2010), we set up a genetic screen to search for spontaneous mutations that suppressed the defective biofilm phenotype of a *B. subtilis ΔslrR* mutant (YC131). The defective biofilm phenotype of the *ΔslrR* mutant is manifested as an inability to form robust floating pellicles (Chu et al., 2008; Chai et al., 2010). Briefly, the *ΔslrR* strain was inoculated into liquid MSgg medium in 6-well plates and incubated at 30°C. After 48 hr, pellicle formation was examined visually. In some wells, robust pellicles appeared possibly due to a second, suppressor mutation elsewhere in the genome. Cells from those wells were picked and streaked out on fresh LB agar plates to isolate single colonies. Cells from the single colonies were then tested for altered colony morphology on solid MSgg medium to confirm the suppressor phenotype. Similar genetic screens in previous studies (Kearns et al., 2005; Chai et al., 2010) had established that mutations in the *sinR* gene could suppress the defective biofilm phenotype of the *ΔslrR* mutant. Therefore, we isolated genomic DNA from the putative suppressor mutants, amplified the *sinR* gene by PCR, and then sequenced the *sinR* locus. Once a mutation in the *sinR* gene was confirmed by sequencing, the same mutation was reconstituted in the wild type background (3610) following a previous protocol (Chai et al., 2010), and assayed for alteration in colony morphology on solid MSgg medium.

### Bacterial growth for ribosome profiling

For ribosome profiling during biofilm formation, fresh colonies were inoculated into 8 ml of MSgg liquid medium and grown for 12 hr at 30°C, 200 rpm. Saturated cultures were diluted 1:1000 into 200 ml aliquots of fresh MSgg medium and shaken in a 1L flask at 30°C, 200 rpm. For exponential-phase ribosome profiling (Figure 2A), cultures were harvested at OD_600_ = 0.6. For ribosome profiling during biofilm entry (Figure 2B), cultures were harvested at OD_600_ = 1.4. For the serine repletion experiment (Figure 4), serine was added to a final concentration of 2.5 mM at OD_600_ = 1.4 and harvested after 30 min at 30°C, 200 rpm. For the serine starvation experiment (Figure 3B,C), pre-cultures were grown in MSgg medium supplemented with 5 mM serine and then diluted into 200 ml of the same medium. At an OD_600_ = 0.6, the cultures were filtered and re-suspended either in MSgg medium (starvation) or in MSgg medium with 5 mM serine (control), and harvested after 60 min at 30°C, 200 rpm (Figure 3D).

### Western blotting

Cultures were grown with shaking at 37°C, and 14 ml culture aliquots were harvested at an OD_600_ between 2.0 and 2.5. Cell pellets were collected by centrifugation and washed once with 10 ml of lysis buffer (20 mM Tris-HCl, 200 mM NaCl, 1 mM EDTA pH 7.4). Pellets were resuspended in 1.2 ml lysis buffer and incubated with 20 µg/ml of lysozyme (Sigma) for 1 hr on ice. Cells were further lysed by sonication. Cell debris was removed by centrifugation (14,000 rpm, 30 min, 4°C). The concentration of total protein in the lysates was determined by a Bradford assay (Bio-Rad, Hercules, CA). Samples for SDS-PAGE were prepared in Laemmli buffer normalized to equal protein concentration. Samples were ran on an NuPAGE 12% gel (Invitrogen, Carlsbad, CA, 1.0 mm, Bis/Tris, 200 V, ∼50 min) and transferred to a PVDF membrane (Millipore, Billerica, MA) at 100 V for 1 hr. The membrane was blocked in 5% milk-TPBS for 1 hr, and then incubated with anti-SinR antibody (1:2500, polyclonal) and anti-SigA (1:100,000, polyclonal) overnight. The membranes were washed 3 times in TPBS for 5 min each. Blots were incubated with goat anti-rabbit secondary antibody conjugated to Horseradish peroxidase (1:10,000, Bio-Rad). Blots were washed three times in TPBS, and developed with SuperSignal West Dura chemiluminescent substrate (Thermo, Waltham, MA) and imaged on a gel-doc (Bio-Rad). Densitometry analysis of Western blot images was performed using ImageJ software (NIH, http://rsbweb.nih.gov/ij/). Rectangles were drawn around each distinct band and the average pixel intensity in this rectangle was calculated, followed by subtraction of background pixel intensity from an identical rectangle drawn in a region without any band. These background subtracted values were used for calculating SinR/SigA, SlrR/SigA and SinR / SlrR ratios in Figure 1.

### YFP fluorescence measurements

A previously published measurement protocol was used (Subramaniam et al., 2013). Fresh colonies were inoculated into 1 ml of MSgg liquid medium with 5 mM serine and grown overnight in deep 96-well plates at 30°C, 1400 rpm. In the morning, saturated cultures were diluted 1:100 into 1 ml of MSgg medium with 800 µM serine and 400 µM serine methyl-ester and shaken at 30°C, 1350 rpm for 3 hr. Three aliquots of 150 µl from each culture was pipetted into three wells of three 96-well plates (3799, Costar, Corning, NY). Wallac Victor2 plate reader (PerkinElmer, Waltham, MA) was used to monitor cell density (absorbance at 600 nm) and YFP expression (fluorescence, excitation 504 nm and emission 540 nm). Each plate was read every 15 min using a robotic system (Caliper Life Sciences, Hopkinton, MA) and shaken at 1000 rpm in between readings (Variomag Teleshake shaker, Daytona Beach, FL). 30°C and 60% relative humidity was maintained throughout the experiment.

### Ribosome profiling and mRNA quantification

Ribosome profiling protocol was adapted from published literature (Ingolia et al., 2009; Oh et al., 2011; Ingolia et al., 2012) with minor modifications as described below. Briefly, 200 ml of bacterial culture was harvested by filtration. The filter was immediately inserted into a 50 ml conical tube, flash frozen in liquid nitrogen, and stored at −80°C until further processing. Frozen cells were re-suspended in 8 ml of polysome resuspension buffer (20 mM Tris pH 8.0, 10 mM MgCl_2_, 100 mM NH_4_Cl, and 100 µg ml^−1^ Chloramphenicol). Re-suspended cells were pelleted by centrifugation (3000 g, 4°C, 5 min) and the supernatant was discarded. The cell pellet was re-suspended in 500 µl of polysome lysis buffer (1X polysome resuspension buffer, 5 mM CaCl_2_, 0.4% TritonX-100, 0.1% NP-40, and 100 U/ml RNase-free DNase [04716728001; Roche]), and transferred to an ice-cold 1.5 ml tube containing 500 µl of 0.2–0.3 µm acid-washed glass beads (G1277; Sigma). Cells were lysed by vortexing at maximum speed on a vortexer in a 4°C room (Vortex Genie 2, 10 × 30 s with 1 min cooling on ice in between). The lysate was clarified by centrifugation (20,000 g, 4°C, 10 min) and the supernatant was transferred to a fresh 1.5 ml tube. 500 µg of total RNA (A_260_ units) was digested (25°C, 1400 rpm, 60 min, 150 µl vol) with 2 U/µg of Micrococcal nuclease (LS004797; Worthington, Lakewood, NJ). The digestion was quenched with 1.5 µl of 0.5 M EGTA, loaded on top of a 10–50% sucrose gradient and ultra-centrifuged in a SW41 rotor (35000 rpm, 4°C, 150 min). Monosomes collected by gradient fractionation (Biocomp Instruments, Canada).

For total RNA extraction, 100 µl of polysome lysate was mixed with 400 µl of RNA extraction buffer (0.3 M sodium acetate, 10 mM EDTA, pH 4.5) and the aqueous phase was extracted twice with phenol-chloroform and once with chloroform. RNA was precipitated with an equal volume of isopropanol. The pellet was washed with 70% ice-cold ethanol and re-suspended in 100 µl of 10 mM Tris pH 7.0. 10 µg of total RNA was DNase-treated and mRNA enriched using the Microbe Express kit (Invitrogen). mRNA was fragmented by heating at 95°C with a bicarbonate buffer (Ingolia et al., 2009) for 20 min.

Collected monosome fractions were purified using the same phenol-chloroform method as used for total RNA extraction above. Monosomes and fragmented mRNA were then used for small RNA sequencing library preparation. Size selection, dephosphorylation, polyadenylation, reverse-transcription, circularization and PCR amplification were performed using the same protocol as in (Ingolia et al., 2009). An rRNA subtraction step was carried out between the circularization and PCR amplification steps using the same protocol as in (Oh et al., 2011). Typically, several samples were multiplexed for sequencing in an Illumina HiSeq sequencer such that at least 1 million reads were obtained for each sample.

### Deep-sequencing data analysis

Deep-sequencing data analysis was carried out in Bash and Python programming languages, and performed on the Harvard research computing Odyssey cluster. Main steps are summarized below and shown schematically in Figure 2—figure supplement 1.

1. Each 50 nt single-end read was polyA-trimmed by identifying 10 or greater number of adjacent adenines and discarding all nucleotides starting from −1nt of the polyA run. The first 5^′^ nt of the read was also discarded since its identity was ambiguous in several reads. PolyA-trimmed reads were first aligned against all non-coding RNAs in the *B. subtilis* genome using bowtie aligner (ver. 0.12.7, Langmead et al., 2009).
2. The non-coding RNA sequences were downloaded from NCBI (NC_000964.frn).
3. Reads that did not align to non-coding RNAs were aligned against the whole *B. subtilis* genome using bowtie aligner. The *B. subtilis* genome was downloaded from NCBI (NC_000964.fna). Only reads that had less than three mismatches with the reference genome were considered for further analysis.
4. Reads that aligned to the *B. subtilis* genome were further trimmed by 8 nt from each end to approximate the ribosome A-site coordinate. The remaining sequence was normalized by its length and assigned to the corresponding genomic coordinate, and this value was designated as the read density at this genomic coordinate during further downstream analyses.
5. Average ribosome and mRNA density for a single gene was calculated by summing the read density between the start and stop codon, and then normalizing by the length of the gene.
6. Fold-change in ribosome density for a single gene between two samples was calculated by taking the log2 of the ratio of average ribosome density between the two samples for that gene. The median value of this log2 fold-change across all genes that received a minimum of 100 reads in at least one of the two samples was then subtracted from the fold-change value for each gene. This median-subtracted log2 fold change in reported throughout this work. We note that the average ribosome density on any gene is directly proportional to the corresponding mRNA level in the absence of specific translational regulation. Hence fold-changes in ribosome density (such as the one shown in Figure 3D) primarily reflect fold-changes in mRNA level.
7. To calculate the ribosome and mRNA density at individual codons,
  - The start codon was treated as a single separate codon irrespective of its identity.
  - Only genes with average ribosome density of at least 1 read per codon were considered.
  - The ribosome density at the first nucleotide of each codon was assigned as the ribosome density at that codon. For each of the 64 codons and the start codon, read density at the first nucleotide of the codon was averaged across all occurrences of that codon in a single gene and then normalized by the average ribosome density for that gene. Hence a codon without over- or under- representation of ribosome or mRNA density will have a density value equal to 1.
  - Genome-wide ribosome and mRNA density was calculated as the median of the individual gene read density from Step (3) across all genes that pass the threshold of Step (2).
  - Ribosome profiling measurement resulted in a high ribosome density at start and stop codons. It is unclear whether this increase is a true biological signal or caused by the measurement protocol (Ingolia et al., 2011). Hence these codons were excluded from the ribosome density plots shown in Figure 2A,B and 3C. However, the increased ribosome density at these codons resulted in a concomitant decrease in ribosome density at the remaining codons due to normalization by the average ribosome density for each gene (which included the start and stop codons).

### Codon usage analysis

Codon Adpatiaton Index (CAI) was calculated according to the original prescription of Sharp and Li (Sharp and Li, 1987). For this calculation, 68 genes that had an annotation as ‘ribosomal’ were used as the reference set of highly expressed genes. The annotations file used for this analysis was downloaded from NCBI (NC_000964.ptt). The p value for the higher frequency of TCN codons in *sinR* was calculated by assuming a binomial distribution of TCN and AGC/AGT codons. The p value represents the binomial probability that there are 10 or more TCN codons in the *sinR* gene (12 serine codons total) given the genome-wide frequency of serine codons (0.66 for TCN codons and 0.34 for AGC/AGT codons).

## Additional files

Supplementary file 1. Lists of strains, plasmids, and primers. **DOI:** http://dx.doi.org/10.7554/eLife.01501.014

### Major dataset

The following dataset was generated:

SubramaniamAR, DeLougheryA, BradshawN, ChenY, O’SheaE, LosickR, ChaiY, 2013, A Serine Sensor for Multicellularity in a Bacterium, ; http://www.ncbi.nlm.nih.gov/geo/query/acc.cgi?acc=GSE50870, Publicly available at NCBI GEO (http://www.ncbi.nlm.nih.gov/geo/).

## References

[bib1] Andersson SG Kurland CG 1990 Codon preferences in free-living microorganisms. Microbiological Reviews 54:198–210 2194095 10.1128/mr.54.2.198-210.1990 PMC372768

[bib2] Beauregard PB Chai Y Vlamakis H Losick R Kolter R 2013 Bacillus subtilis biofilm induction by plant polysaccharides. Proceedings of the National Academy of Sciences of the United States of America 110:E1621–E1630. 10.1073/pnas.1218984110 23569226 PMC3637697

[bib3] Bernstein JA Khodursky AB Lin P-H Lin-Chao S Cohen SN 2002 Global analysis of mRNA decay and abundance in *Escherichia coli* at single-gene resolution using two-color fluorescent DNA microarrays. Proceedings of the National Academy of Sciences of the United States of America 99:9697–9702. 10.1073/pnas.112318199 12119387 PMC124983

[bib4] Branda SS González-Pastor JE Ben-Yehuda S Losick R Kolter R 2001 Fruiting body formation by Bacillus subtilis. Proceedings of the National Academy of Sciences of the United States of America 98:11621–11626. 10.1073/pnas.191384198 11572999 PMC58779

[bib5] Chai Y Chu F Kolter R Losick R 2008 Bistability and biofilm formation in *Bacillus subtilis*. Molecular Microbiology 67:254–263. 10.1111/j.1365-2958.2007.06040.x 18047568 PMC2430929

[bib6] Chai Y Kolter R Losick R 2009 A widely conserved gene cluster required for lactate utilization in *Bacillus subtilis* and its involvement in biofilm formation. Journal of Bacteriology 191:2423–2430. 10.1128/JB.01464-08 19201793 PMC2668416

[bib7] Chai Y Norman T Kolter R Losick R 2010 An epigenetic switch governing daughter cell separation in *Bacillus subtilis*. Genes and Development 24:754–765. 10.1101/gad.1915010 20351052 PMC2854391

[bib8] Chai Y Norman T Kolter R Losick R 2011 Evidence that metabolism and chromosome copy number control mutually exclusive cell fates in *Bacillus subtilis*. The EMBO Journal 30:1402–1413. 10.1038/emboj.2011.36 21326214 PMC3094124

[bib9] Charneski CA Hurst LD 2013 Positively charged residues are the major determinants of ribosomal velocity. PLOS Biol 11:e1001508. 10.1371/journal.pbio.1001508 23554576 PMC3595205

[bib10] Chen Y Cao S Chai Y Clardy J Kolter R Guo JH Losick R 2012 A *Bacillus subtilis* sensor kinase involved in triggering biofilm formation on the roots of tomato plants. Molecular Microbiology 85:418–430. 10.1111/j.1365-2958.2012.08109.x 22716461 PMC3518419

[bib11] Chu F Kearns DB Branda SS Kolter R Losick R 2006 Targets of the master regulator of biofilm formation in *Bacillus subtilis*. Molecular Microbiology 59:1216–1228. 10.1111/j.1365-2958.2005.05019.x 16430695

[bib12] Chu F Kearns DB McLoon A Chai Y Kolter R Losick R 2008 A novel regulatory protein governing biofilm formation in *Bacillus subtilis*. Molecular Microbiology 68:1117–1127. 10.1111/j.1365-2958.2008.06201.x 18430133 PMC2430766

[bib39] Cruz Ramos H Hoffmann T Marino M Nedjari H Presecan-Siedel E Dreesen O Glaser P Jahn D 2000 Fermentative metabolism of *Bacillus subtilis*: physiology and regulation of gene expression. Journal of Bacteriology 182:3072–3080. 10.1128/JB.182.11.3072-3080.2000 10809684 PMC94491

[bib13] Dittmar KA Sorensen MA Elf J Ehrenberg M Pan T 2005 Selective charging of tRNA isoacceptors induced by amino-acid starvation. EMBO Reports 6:151–157. 10.1038/sj.embor.7400341 15678157 PMC1299251

[bib14] Doi RH Kaneko I Goehler B 1966 Regulation of a serine transfer RNA of *Bacillus subtilis* under two growth conditions. Proceedings of the National Academy of Sciences of the United States of America 56:1548–1551. 10.1073/pnas.56.5.1548 4961689 PMC220027

[bib15] Elf J Nilsson D Tenson T Ehrenberg M 2003 Selective charging of tRNA isoacceptors explains patterns of codon usage. Science 300:1718–1722. 10.1126/science.1083811 12805541

[bib16] Gagnon Y Breton R Putzer H Pelchat M Grunberg-Manago M Lapointe J 1994 Clustering and co-transcription of the *Bacillus subtilis* genes encoding the aminoacyl-tRNA synthetases specific for glutamate and for cysteine and the first enzyme for cysteine biosynthesis. Journal of Biological Chemistry 269:7473–7482 7510287

[bib17] Hayes CS Sauer RT 2003 Cleavage of the A site mRNA codon during ribosome pausing provides a mechanism for translational quality control. Molecular Cell 12:903–911. 10.1016/S1097-2765(03)00385-X 14580341

[bib18] Henkin TM Yanofsky C 2002 Regulation by transcription attenuation in bacteria: how RNA provides instructions for transcription termination/antitermination decisions. Bioessays 24:700–707. 10.1002/bies.10125 12210530

[bib19] Ingolia NT Ghaemmaghami S Newman JRS Weissman JS 2009 Genome-wide analysis in vivo of translation with nucleotide resolution using ribosome profiling. Science 324:218–223. 10.1126/science.1168978 19213877 PMC2746483

[bib20] Ingolia NT Lareau LF Weissman JS 2011 Ribosome profiling of mouse embryonic stem cells reveals the complexity and dynamics of mammalian proteomes. Cell 147:789–802. 10.1016/j.cell.2011.10.002 22056041 PMC3225288

[bib21] Ingolia NT Brar GA Rouskin S McGeachy AM Weissman JS 2012 The ribosome profiling strategy for monitoring translation in vivo by deep sequencing of ribosome-protected mRNA fragments. Nature Protocols 7:1534–1550. 10.1038/nprot.2012.086 PMC3535016 22836135

[bib22] Jiang M Shao W Perego M Hoch JA 2000 Multiple histidine kinases regulate entry into stationary phase and sporulation in *Bacillus subtilis*. Molecular Microbiology 38:535–542. 10.1046/j.1365-2958.2000.02148.x 11069677

[bib23] Kearns DB Chu F Branda SS Kolter R Losick R 2005 A master regulator for biofilm formation by *Bacillus subtilis*. Molecular Microbiology 55:739–749. 10.1111/j.1365-2958.2004.04440.x 15661000

[bib24] Keiler KC Waller PRH Sauer RT 1996 Role of a peptide tagging system in degradation of proteins synthesized from damaged messenger RNA. Science 271:990–993. 10.1126/science.271.5251.990 8584937

[bib25] Kolodkin-Gal I Elsholz AK Muth C Girguis PR Kolter R Losick R 2013 Respiration control of multicellularity in *Bacillus subtilis* by a complex of the cytochrome chain with a membrane-embedded histidine kinase. Genes and Development 27:887–899. 10.1101/gad.215244.113 23599347 PMC3650226

[bib26] Kolter R Greenberg EP 2006 Microbial sciences: the superficial life of microbes. Nature 441:300–302. 10.1038/441300a 16710410

[bib27] Kudla G Murray AW Tollervey D Plotkin JB 2009 Coding-sequence determinants of gene expression in *Escherichia coli*. Science 324:255–258. 10.1126/science.1170160 19359587 PMC3902468

[bib28] Langmead B Trapnell C Pop M Salzberg SL 2009 Ultrafast and memory-efficient alignment of short DNA sequences to the human genome. Genome Biol 10:R25. 10.1186/gb-2009-10-3-r25 19261174 PMC2690996

[bib29] Li G-W Oh E Weissman JS 2012 The anti-Shine-Dalgarno sequence drives translational pausing and codon choice in bacteria. Nature 484:538–541. 10.1038/nature10965 22456704 PMC3338875

[bib30] Liebs P Riedel K Graba JP Schrapel D Tischler U 1988 Formation of some extracellular enzymes during the exponential growth of *Bacillus subtilis*. Folia Microbiologica 33:88–95. 10.1007/BF02928073 3131218

[bib31] López D Fischbach MA Chu F Losick R Kolter R 2009 Structurally diverse natural products that cause potassium leakage trigger multicellularity in *Bacillus subtilis*. Proceedings of the National Academy of Sciences of the United States of America 106:280–285. 10.1073/pnas.0810940106 19114652 PMC2629187

[bib32] Lowe TM Eddy SR 1997 tRNAscan-SE: a program for improved detection of transfer RNA genes in genomic sequence. Nucleic Acids Research 25:955–964. 10.1093/nar/25.5.0955 9023104 PMC146525

[bib33] Marin FT 1976 Regulation of development in *Dictyostelium discoideum*: I. Initiation of the growth to development transition by amino acid starvation. Developmental Biology 48:110–117. 10.1016/0012-1606(76)90050-6 942703

[bib34] Molle V Fujita M Jensen ST Eichenberger P González-Pastor JE Liu JS Losick R 2003 The Spo0A regulon of *Bacillus subtilis*. Molecular Microbiology 50:1683–1701. 10.1046/j.1365-2958.2003.03818.x 14651647

[bib35] Norman T Lord N Paulsson J Losick R 2013 Memory and modularity in cell-fate decision making. Nature 503:481–486. 10.1038/nature12804 24256735 PMC4019345

[bib36] Oh E Becker AH Sandikci A Huber D Chaba R Gloge F Nichols RJ Typas A Gross CA Kramer G Weissman JS Bukau B 2011 Selective ribosome profiling reveals the cotranslational chaperone action of trigger factor in vivo. Cell 147:1295–1308. 10.1016/j.cell.2011.10.044 22153074 PMC3277850

[bib37] Plotkin JB Kudla G 2010 Synonymous but not the same: the causes and consequences of codon bias. Nature Reviews Genetics 12:32–42. 10.1038/nrg2899 PMC3074964 21102527

[bib38] Prüss BM Nelms JM Park C Wolfe AJ 1994 Mutations in NADH:ubiquinone oxidoreductase of *Escherichia coli* affect growth on mixed amino acids. Journal of Bacteriology 176:2143–2150 8157582 10.1128/jb.176.8.2143-2150.1994 PMC205332

[bib40] Sambrook J 2001 Molecular cloning: a laboratory Manual. 3rd ed Long Island, NY: Cold Spring Harbor Laboratory Press

[bib41] Sezonov G Joseleau-Petit D D’Ari R 2007 *Escherichia coli* physiology in Luria-Bertani broth. Journal of Bacteriology 189:8746–8749. 10.1128/JB.01368-07 17905994 PMC2168924

[bib42] Sharp PM Li WH 1987 The Codon Adaptation Index–a measure of directional synonymous codon usage bias, and its potential applications. Nucleic Acids Research 15:1281–1295. 10.1093/nar/15.3.1281 3547335 PMC340524

[bib43] Shemesh M Kolter R Losick R 2010 The biocide chlorine dioxide stimulates biofilm formation in *Bacillus subtilis* by activation of the histidine kinase KinC. Journal of Bacteriology 192:6352–6356. 10.1128/JB.01025-10 20971918 PMC3008516

[bib44] Subramaniam AR Pan T Cluzel P 2013 Environmental perturbations lift the degeneracy of the genetic code to regulate protein levels in bacteria. Proceedings of the National Academy of Sciences of the United States of America 110:2419–2424. 10.1073/pnas.1211077110 23277573 PMC3568297

[bib45] Sundrud MS Koralov SB Feuerer M Calado DP Kozhaya AE Rhule-Smith A Lefebvre RE Unutmaz D Mazitschek R Waldner H Whitman M Keller T Rao A 2009 Halofuginone inhibits TH17 cell differentiation by activating the amino acid starvation response. Science 324:1334–1338. 10.1126/science.1172638 19498172 PMC2803727

[bib46] Vlamakis H Chai Y Beauregard P Losick R Kolter R 2013 Sticking together: building a biofilm the *Bacillus subtilis* way. Nature Reviews Microbiology 11:157–168. 10.1038/nrmicro2960 PMC3936787 23353768

[bib47] Wach A 1996 PCR-synthesis of marker cassettes with long flanking homology regions for gene disruptions in *S. cerevisiae*. Yeast 12:259–265. 10.1002/(SICI)1097-0061(19960315)12:3<259::AID-YEA901>3.0.CO;2-C 8904338

[bib48] Wang J Alexander P Wu L Hammer R Cleaver O McKnight SL 2009 Dependence of mouse embryonic stem cells on threonine catabolism. Science 325:435–439. 10.1126/science.1173288 19589965 PMC4373593

[bib49] West AH Stock AM 2001 Histidine kinases and response regulator proteins in two-component signaling systems. Trends in Biochemical Sciences 26:369–376. 10.1016/S0968-0004(01)01852-7 11406410

